# Novel Genetic and Molecular Pathways in Pulmonary Arterial Hypertension Associated with Connective Tissue Disease

**DOI:** 10.3390/cells10061488

**Published:** 2021-06-13

**Authors:** Ignacio Hernandez-Gonzalez, Jair Tenorio-Castano, Nuria Ochoa-Parra, Natalia Gallego, Carmen Pérez-Olivares, Mauro Lago-Docampo, Julian Palomino Doza, Diana Valverde, Pablo Lapunzina, Pilar Escribano-Subias

**Affiliations:** 1 Department of Cardiology, Hospital Universitario Río Hortega, 47012 Valladolid, Spain; 2 Institute of Medical and Molecular Genetics (INGEMM)-IdiPAZ, Hospital Universitario La Paz-UAM, Paseo de La Castellana, 261, 28046 Madrid, Spain; jairantonio.tenorio@gmail.com (J.T.-C.); nataliagallegozazo@gmail.com (N.G.); pablo.lapunzina@salud.madrid.org (P.L.); 3 CIBERER, Centro de Investigación Biomédica en Red de Enfermedades Raras, ISCIII, Melchor Fernández Almagro Street, 3, 28029 Madrid, Spain; 4 ITHACA, European Reference Network on Rare Congenital Malformations and Rare Intellectual Disability, Hospital Universitario La Paz, 28046 Madrid, Spain; 5 Unidad Multidisciplinar de Hipertensión Pulmonar, Servicio de Cardiología, Hospital Universitario 12 de Octubre, 28041 Madrid, Spain; nuriaochoaparra@hotmail.com (N.O.-P.); carmenperezolivaresd@gmail.com (C.P.-O.); julianpalomino@gmail.com (J.P.D.); pilar.escribano.subias@gmail.com (P.E.-S.); 6 CINBIO, Universidade de Vigo, 36310 Vigo, Spain; maurolagodocampo@gmail.com (M.L.-D.); dianaval@uvigo.es (D.V.); 7 Instituto de Investigación Sanitaria Galicia Sur (IIS Galicia Sur), SERGAS-UVIGO, 36312 Vigo, Spain; 8 Unidad de Miocardiopatías Familiares, Servicio de Cardiología, Hospital Universitario 12 de Octubre, 28041 Madrid, Spain; 9 CIBERCV, Centro de Investigación Biomédica en Red de Enfermedades Cardiovasculares, ISCIII, 28029 Madrid, Spain

**Keywords:** PAH, BMP signalling, genetics, immunity

## Abstract

Pulmonary Arterial Hypertension (PAH) is a severe complication of Connective Tissue Disease (CTD), with remarkable morbidity and mortality. However, the molecular and genetic basis of CTD-PAH remains incompletely understood. This study aimed to screen for genetic defects in a cohort of patients with CTD-PAH, using a PAH-specific panel of 35 genes. During recruitment, 79 patients were studied, including 59 Systemic Sclerosis patients (SSc) and 69 females. Disease-associated variants were observed in nine patients: 4 pathogenic/likely pathogenic variants in 4 different genes (*TBX4, ABCC8, KCNA5* and *GDF2/BMP9*) and 5 Variants of Unknown Significance (VUS) in 4 genes (*ABCC8, NOTCH3, TOPBP1* and *CTCFL*). One patient with mixed CTD had a frameshift pathogenic variant in *TBX4*. Two patients with SSc-PAH carried variants in *ABCC8*. A patient diagnosed with Systemic Lupus Erythematous (SLE) presented a pathogenic nonsense variant in *GDF2/BMP9*. Another patient with SSc-PAH presented a pathogenic variant in *KCNA5*. Four patients with SSc-PAH carried a VUS in *NOTCH1, CTCFL, CTCFL* and *TOPBP1*, respectively. These findings suggest that genetic factors may contribute to Pulmonary Vascular Disease (PVD) in CTD patients.

## 1. Introduction

Pulmonary Arterial Hypertension (PAH) is a feared complication of Connective Tissue Diseases (CTD), with remarkable morbidity and mortality [1]. Systemic sclerosis (SSc) is most commonly associated with PAH, but it can be present in other CTD such as Systemic Lupus Erythematous (SLE) or Mixed CTD (MCTD) [2]. CTD-associated PAH (CTD-PAH) is present in up to 12% of patients with SSc and it is one of the leading disease-related causes of death [3]. Moreover, CTD-PAH represents 15–30% of cases in PAH registries [4,5]. Despite major advances in PAH therapy, survival in CTD-PAH remains poor, with a three-year survival of 40–50% [4].

To date, 12 genes have been associated with PAH with a high level of evidence, and 5 have been associated with a low level of evidence [6]. Furthermore, high-throughput sequencing (HTS) technologies have led to the identification of novel associated genes [7]. The main gene involved in PAH encodes the bone morphogenic protein receptor type 2 (*BMPR2*), a receptor belonging to the transforming growth factor beta (TGF-β) superfamily [8]. Other genes have also been identified: potassium channel genes (*KCNK3*, *KCNA5*, *ABCC8*), T-box transcription factor 4 (*TBX4*), and other genes in the TGF-β/BMP signaling pathway (*BMP9/GDF2*, *SMAD1*, *SMAD4*, *SMAD9*, *BMPR1B*) [6]. Previous studies have demonstrated that rare coding mutations are present in ~80% of familial forms and ~20% of sporadic cases [8].

Currently, the molecular and genetic basis of PAH in CTD has not been fully addressed. Previous studies have suggested that genetic factors may play a significant role in the development of Pulmonary Vascular Disease (PVD) in other conditions, such as congenital heart disease [9]. However, the role of genetic abnormalities in CTD-associated PVD remains unclear.

This study aimed to screen for genetic defects in a cohort of patients with CTD-PAH.

## 2. Materials and Methods

### 2.1. Study Patients

Since November 2011, genetic testing has been offered to all patients with idiopathic, hereditable and associated forms of PAH, and Pulmonary Venooclusive Disease (PVOD), included in the Spanish Registry of Pulmonary Arterial Hypertension (REHAP). A full list of REHAP centers and investigators is provided in the Supporting Information (See Table S1).

Pulmonary Arterial Hypertension was defined according to the 2015 ERS/ESC Guidelines for the Diagnosis and Treatment of Pulmonary Hypertension [10]. Routine diagnostic workup included medical history, physical examination, 6-min walking test (6MWT), echocardiogram, multidetector computed tomography (MDCT), ventilation/perfusion lung scan, pulmonary function tests (PFT), and screening of connective tissue disease, HIV infection and portal hypertension. Right Heart Catheterism (RHC) at diagnosis includes Right Atrium Pressure, Mean Pulmonary Artery Pressure, Pulmonary Wedge Pressure, Cardiac Output, Cardiac Index and Pulmonary Vascular Resistance. Pulmonary vasoreactivity testing was performed in Idiopathic PAH (IPAH), Hereditable PAH (HPAH) and drug-induced PAH. Routine diagnostic workup included medical history, physical examination, 6-min Walking Test (6MWT), echocardiogram, Multidetector Computed Tomography (MDCT), ventilation/perfusion lung scan, pulmonary function tests (PFT), and screening of Connective Tissue Disease, HIV infection and Portal Hypertension. PFT included the diffusing capacity for carbon monoxide (DLCO), which was considered moderately reduced when DLCO 43–62% of predicted values and severely reduced when DLCO < 43% of predicted values [11]. Therapeutic management is left to the discretion of individual physicians.

All patients or legal tutors included in the analysis gave their written informed consent and the project was approved by the ethical committee for scientific research of the participant centers. We obtained written parental consent from the parents or guardians of minors included in this study.

### 2.2. Molecular Analysis

A PAH-specific HTS panel of 35 genes was designed, including all PAH-associated genes at that date with a variable level of evidence [6]. Review, classification and interpretation of variants were carried out according to the American College of Medical Genetics and Genomics guidelines [12]. The ethical principles of the European Board of Medical Genetics and the 2015 ERS/ESC guidelines for the diagnosis and treatment of pulmonary hypertension offer accurate information on the range of options available to make informed decisions, and allow equal access to genetic counseling and testing [10]. Pre- and post-test genetic counseling was provided. In the pre-test visit, family history information was collected, but only probands were studied. When a positive result was observed, a genetic study was offered to first degree relatives where available. Cascade or co-segregation genetic tests were also performed. When an unaffected carrier was identified, a complete diagnostic was performed, including electrocardiogram, echocardiogram, N-terminal pro-brain natriuretic peptide (NT-proBNP) and 6 Minute Walking Test. This evaluation is periodically repeated. When a sustained suspicion of early-stage PAH was observed, RHC was performed to rule out the condition.

## 3. Results

**Figure 1:**
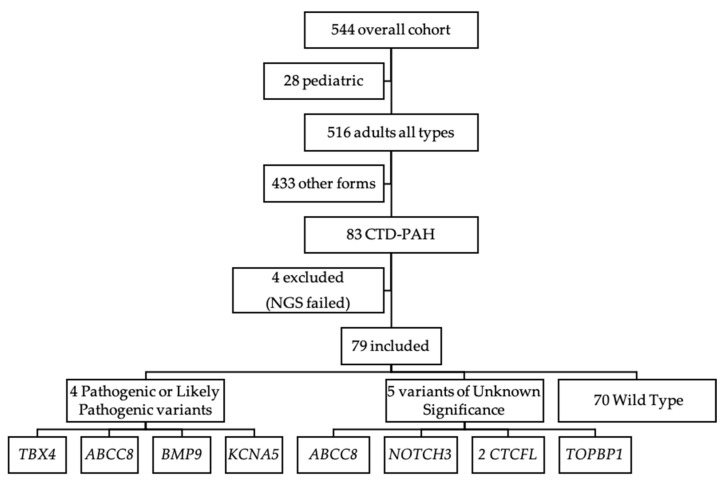
Patient selection flow chart. PAH: Pulmonary Arterial Hypertension; CTD-PAH: Pulmonary Arterial Hypertension Associated with Connective Tissue Disease.

During patients’ enrolling, 79 CTD-PAH patients were recruited: 59 SSc, 11 Systemic Lupus Erythematous (SLE) and 9 other CTD (Figure 1). Baseline characteristics are shown in Table 1. Sixty-nine patients were female, mean age was 55.6 ± 1.9 years, mean pulmonary vascular resistance (PVR) was 8.6 ± 0.5 wood units (WU) and mean diffusing capacity of the lung for carbon monoxide (DLCO) was 47.5 ± 2% of predicted value.

Disease-associated variants were observed in nine patients. Four of them were classified as pathogenic or likely pathogenic in four different genes (*TBX4*, *ABCC8*, *KCNA5* and *GDF2/BMP9*), and five as variants of unknown significance (VUS) in four genes (*ABCC8*, *NOTCH3*, *TOPBP1* and *CTCFL*). Clinical characteristics of patients with pathogenic or likely pathogenic variants and variant analyses are shown in Table 2 and Table 3, respectively.

Patient 1 is a Caucasian female with Mixed CTD, diagnosed with PAH at 58 years of age. She has a frameshift pathogenic variant in *TBX4*: (NM_018488.3): c.1112dupC:p.(Pro372Serfs*14). A Pulmonary function test (PFT) at diagnosis ruled out interstitial lung disease (ILD), but a reduction in DLCO was observed (61% of predicted value). Small Patella Syndrome was also ruled out. Up-front oral combination therapy was prescribed. After six years of follow-up, she has a low-risk profile under double oral combination therapy.

Two patients carry variants in *ABCC8*. Patient 2 is a Caucasian female with SSc, diagnosed with PAH at 27 years of age. She carries a splicing variant in *ABCC8*: (NM_000352.6): c.2694+1G>A, classified as likely pathogenic. Her mother was diagnosed with PAH, associated with a repaired atrial septal defect, at 61 years of age. In the genetic testing, no variants were observed in *ABCC8* or other PAH genes. ILD was also ruled out in patient 2. However, a mild reduction in diffusion capacity was observed at diagnosis (DLCO 71% of predicted value). Monotherapy was initiated. During follow-up, goal-oriented PAH therapy was applied, and risk profile was assessed periodically. Eighteen years after diagnosis, she presents a low-risk profile under triple combination therapy, including systemic prostanoids. In this time, diffusion capacity progressively worsened (current DLCO 45% of predicted value), without signs of ILD. Patient 3 is a Caucasian male, with clinical suspicion of PVOD associated with SSc and HIV infection. PAH was diagnosed at 57 years of age. He presented a missense variant in *ABCC8* (NM_000352.6):c.298G>A p.(Glu100Lys), located in a gating regulatory region, and classified as VUS. His sister was also diagnosed with PVOD associated with SSc at 48 years of age. Referred to the lung transplant unit, she died on the waiting list. No blood or tissue samples are available for histological or genetic analysis. In patient 3, DLCO at diagnosis was 22% of predicted value. MDCT showed the typical PVOD triad, consisting of ground grass opacification, interlobular septal thickening or mediastinal lymphadenopathy. He also had respiratory insufficiency when resting and a significant drop in oxygen desaturation during exercise. Although referred to the lung transplant unit, he was not eligible due to advanced age and comorbidities. The clinical course was progressive, and he died 4.5 years after the diagnosis.

Patient 4 is a Latin American female with SLE, diagnosed with PAH at 25 years of age. She presented a nonsense variant in *GDF2/BMP9*: (NM_016204.4): c.642G>A: (p.Trp214*) which causes the appearance of a premature stop codon, classified as pathogenic. Three years after diagnosis, she presented a low-risk profile under dual oral therapy.

Patient 5 is a Caucasian female with SSc, diagnosed with PAH at 70 years of age. She presented a pathogenic variant in *KCNA5*: (NM_002234.3):c.1685delC(p.Phe563fs*21). During follow-up, goal-oriented PAH therapy was applied. She died 8.5 years after diagnosis due to progressive heart failure.

Another patient with SSc carries a VUS in *NOTCH1*. Three patients show variants in novel PAH-related genes. Patients 9 and 10 had a previous diagnosis of SSc and carry a VUS in *CTCFL*. Patient 11 was diagnosed with SSc-PAH and carries a VUS in *TOPBP1*.

## 4. Discussion

The pathobiology of PAH-CTD remains incompletely understood. On the one hand, it is speculated that this complication may be triggered by immune dysregulation present in CTD [2,13,14]. On the other hand, an imbalance in the TGF-β/BMP axis might also contribute to CTD-PAH development [15,16]. However, despite progress in our knowledge of CTD-PVD, neither genetic theory nor inflammatory theory have been proven. In hereditable and idiopathic forms of PAH, *BMPR2* haploinsufficiency is the most common inherited molecular mechanism [8,17]. However, the penetrance of the disease phenotype is incomplete and additional stimuli are necessary [18]. Female sex is the single most important factor influencing the development of PAH in mutation carriers [19]. Other factors might be genetic (a second variant in another gene), epigenetic or environmental [17,20]. Furthermore, inflammatory cells and their mediator also contribute to pulmonary vascular remodeling in idiopathic forms [21]. Whether the proinflammatory state in CTD is a trigger in genetically susceptible individuals remains unclear [22,23,24,25]. One might speculate that the simultaneous occurrence of genetic and inflammatory factors might explain PAH in CTD [6]. Nevertheless, the genetic basis of CTD-PAH has not been well elucidated to date.

In our CTD-PAH cohort, four patients (5.1%) carried a pathogenic or likely pathogenic variant in a PAH-related gene. Furthermore, VUS were observed in another five patients (6.3%). Functional assays must be performed in order to confirm or discard the possible role of these variants in protein function, and whether this can be related to the phenotype. Some previous studies failed to observe variants in PAH-related genes throughout this population [26]. The most likely explanation for this is that only one or a small number of genes were included. Furthermore, current guidelines for the management of PAH do not recommend genetic testing in associated forms, and most previous studies have excluded them [10]. However, a recent study by Zhu et al. studied 722 CTD-PAH patients [7]. Rare coding variants were observed in 5.26% of the cohort. However, phenotype and clinical information are not provided. For our study, a PAH-specific HTS panel was designed (35 genes), and associated forms of PAH and PVOD were also included. As a result, we also obtained a significant number of variant carriers.

Our study highlights different molecular pathways involved in CTD-PAH. Half of the pathogenic or likely pathogenic variants were located in potassium channel genes (*KCNA5*, *ABCC8*). Only one gene was included in the TGF β pathway (*GDF2/BMP9*) and no variants were observed in *BMPR2*. Another pathogenic variant was present in *TBX*, whose mutations express an ever-expanding phenotype. With this in mind, one might speculate that screening genetic variants may be a practical non-invasive tool to identify high risk CTD patients. Furthermore, it might be especially useful in rare forms of CTD-PAH: early-onset SSc or CTD other than SSc. However, the presence of a genetic defect does not seem to influence clinical course or prognosis.

TGF-β signaling pathway expression and activity is reduced in both idiopathic and hereditable PAH, regardless of the presence of germline mutations [27]. Restoration of the BMPR2 axis is a promising therapy to prevent or treat PAH, by restoring the balance between proliferative and anti-proligerative pathways [13,28,29] Recently, PULSAR trial has demonstrated the benefit of sotatercept in PAH, including a high proportion of CTD-PAH patients. *BMP9/GDF2* is a ligand of the BMP signaling pathway, recently identified as a PAH gene [30]. Furthermore, the application of recombinant BMP9 reversed pulmonary hypertension in animal models [31]. Restoring BMPR2 expression is also a promising treatment target [13,32,33]. The presence of germline mutations in BMP pathway genes suggests that this mechanism might contribute to PVD in CTD patients.

*TBX4* is involved in the regulation of embryonic developmental processes and its haploinsufficiency was classically associated with Small Patella Syndrome. Pathogenic variants in this gene are a common cause of hereditable PAH in infants and children [34,35]. Recently, our group has demonstrated that PAH associated with TBX4 variants shows a wide spectrum of clinical presentations, with overlapping forms of PAH [36]. A severely reduced DLCO was a common finding. However, *TBX4* mutations are not an established cause of CTD-PAH. Thus, the relationship between *TBX4* variants and connective tissue diseases remains unclear. Further investigation is necessary to address this issue.

In our cohort, three patients carry a variant in a potassium channel gene. There is growing evidence supporting the role of potassium channel dysfunction in PAH [37]. *KCNK3* and *KCNA5* are considered to play a predominant role in pulmonary vascular tone [38]. *ABCC8* codifies for SUR1, a subunit of the ATP-sensitive potassium channel that is mostly present in the β pancreatic cells. This explains why pathogenic variants in ABCC8 have been widely related to diabetes mellitus and congenital hyperinsulinism. However, how *ABCC8* can cause PAH remains unclear. Previous studies done by our group and others have observed rare coding mutations in *ABCC8* in idiopathic, familial and CHD-associated PAH [39,40]. However, the role of potassium channels in PAH associated with CTD is not well researched. Further investigation might explain the importance of resting membrane potential in the development of pulmonary vasculopathy in this population.

## 5. Conclusions

These findings suggest that genetic testing might be a useful tool in screening or initial diagnosis work-up of CTD-PAH. The discovery of rare variants in these patients forces us to take a comprehensive approach and provide accurate genetic counseling. Further research is still necessary to confirm these findings and help to provide a personalized medicine approach to these patients.

## Figures and Tables

**Table 1:** Baseline characteristics of 79 CTD patients.

**Female**	69 (87.3%)
Age (years)	55.6 ± 1.9
**Right Heart Catheterism**	
RAP (mmHg)	8.6 ± 0.6
mPAP (mmHg)	42 ± 1.4
PCWP (mmHg)	9.8 ± 0.4
CO (l/min)	4.3 ± 0.1
CI (l/min/m^2^)	2.6 ± 0.1
PVR (UW)	8.6 ± 0.5
SvO2 (%)	65.8 ± 1.5
**Pulmonary Function Test**	
FEV1 (% predicted)	80.5 ± 2.2
FVC (% predicted)	81.7 ± 2.2
TLC (% predicted)	88.5 ± 2.6
DLCO (% predicted)	47.5 ± 2
6MWT (m)	340 ± 16
Exercise O2 Sat (%)	87 ± 1.3
**Functional Class**	
I	2 (2.5%)
II	27 (34.2%)
III	43 (54.4%)
IV	7 (8.9%)
Data are median mean ± SD, or n (%)	

DLCO: Diffusing Capacity of the Lung for Carbon Monoxide; VFC: Vital Forced Capacity; FE1V: Forced Expiratory Volume in 1 s; TLC: Total Lung Capacity; RAP: Right Atrium Pressure; mPAP: mean Pulmonary Artery Pressure; PCWP: Pulmonary Capillary Wedge Pressure; CO: Cardiac Output; CI: Cardiac Index; PVR: Pulmonary Vascular Resistance; WU: Wood Units; 6MWT: Six Minute Walk Test; m: meters; SvO2: Mixed venous oxygen saturation; O2: oxygen.

**Table 2:** Clinical characteristics of patients with pathogenic or likely pathogenic variants.

	Patient 1	Patient 2	Patient 4	Patient 5
Gene	*TBX4*	*ABCC8*	*GDF2*	*KCNA5*
Age PAH diagnosis	58	26	25	70
Age CTD diagnosis	56	28	22	NA
Gender	F	F	F	F
CTD	Mixed CTD	SSc	SLE	SSc
CTD manifestations	Arthritis	Raynaud, digital ulcers	Discoid lupus, enteritis, serositis, poliarthritis	NA
CTD serology	NA	ANA, ACA	ANA, anti-DNA, anti-Sm	NA
CTD treatment	None	Corticosteoids, AZA	Corticosteoids, MTX, cyclophosmamide, hydroxychloroquine	None
mPAP (mmHg)	37	71	30	45
PCWP (mmHg)	4	3	6	8
CI (l/min/m2)		2.25	4.2	4.6
PVR (WU)	12.6	10.2	3.75	4.5
FEV1 (% predicted)	83	102	86	75
TLC or FVC (% predicted)	109	87	103	81
DLCO (% predicted)	61	74		
6MWT (m)	389	463	NA	180
FC	II	III	II	III
Final status	Alive	Alive	Alive	Death
Follow-up (years)	6	18	3.5	8.5

DLCO: Diffusing Capacity of the Lung for Carbon Monoxide; VFC: Vital Forced Capacity; FE1V: Forced Expiratory Volume in 1 s; mPAP: mean Pulmonary Artery Pressure; PCWP: Pulmonary Capillary Wedge Pressure; CO: Cardiac Output; CI: Cardiac Index; WU: Wood Units; PVOD: Pulmonary Venooclusive Disease; SSc: Systemic Sclerosis, SLE: Systemic Erythematous Lupus, CTD: Connective Tissue Disease; HRCT: High-Resolution Computed Tomography; FC: Functional Class; Ex: Exercise; Sat: Saturation; AZA: azathioprine; MTX: methotrexate; NA: not available.

**Table 3:** Variant analysis.

Patient	Gene	Genomic Coordinate (hg19)	cDNA and Protein Location	Exon/Intron	Mutation Type	Population Frequency ^†^	Pathogenicity Predictors ^‡^	ACMG Prediction ^§^	Reference
1	*TBX4*	chr17:59560351dup	NM_018488.2:c.1112dupC:p. (Pro372Serfs*14)	8	frameshift	0	3/3	P	PMID: 32348326
2	*ABCC8*	chr11:17432062C>T	NM_000352.4:c.2694+1G>A	IVS21	splicing	0.000003979	2/2	LP	Lago-Docampo et al.
3	*ABCC8*	chr11:17491762G>A	NM_000352.6:c.298G>A p. (Glu100Lys)	3	missense	0.00007162	2/9	VUS	Lago-Docampo et al.
4	*GDF2*	chr10:48414226C>T	NM_016204.3:c.642G>A:p. (Trp214*)	2	nonsense	0	3/3	P	Tenorio et al.
5	*KCNA5*	chr12:5154998del	NM_002234.3:c.1685delC (p.Phe563fs*21)	1	frameshift	0	2/2	P	Tenorio et al.
6	*NOTCH3*	chr19:15278219C>T	NM_000435.2:c.5203G>A:p. (Glu1735Lys)	29	missense	0.00000409	8/9	VUS	Tenorio et al.
7	*CTCFL*	chr20:56078510G>A	NM_001269041.1:c.1822G>A (p.Glu608Lys)	9	missense	0	1/9	VUS	This study
8	*CTCFL*	chr20:56093935A>C	NM_001269041.1:c.938A>G (p.Tyr313Cys)	4	missense	0	4/9	VUS	This study
9	*TOPBP1*	chr3:133371445T>G	NM_007027.3:c.951T>G (p.Ile317Met)	8	missense	0.0000299	4/9	VUS	This study

^†^ gnomAD exomes, gnomAD genomes, Kaviar, 1000G phase III, ESP. ^‡^ Number of in silico tools that predict pathogenic effect over the total analysed from dbNSFP (MutationTaster, MutationAssessor, FATHMM, FATHMM-MKL, MetaSVM, MetalR, Provean, LRT, SIFT). ^§^ ACMG prediction: P: Pathogenic, LP: Likely Pathogenic, VUS: Variant of Unknown Significance.

## Data Availability

All relevant data are within the manuscript and its Supporting Information files.

## Supplementary Materials

The following are available online at https://www.mdpi.com/article/10.3390/cells10061488/s1, Table S1: Spanish Registry of Pulmonary Arterial Hypertension Centers and Investigators.
