# Supplementary material for: Novel Genetic and Molecular Pathways in Pulmonary Arterial Hypertension Associated with Connective Tissue Disease
Source: Cells. 2021 Jun 13;10(6):1488. doi: 10.3390/cells10061488 (PMC8231632; doi:10.3390/cells10061488)
Supplement: Supplementary file 1 [file cells-10-01488-s001.zip › cells-1231663-supplementary.pdf]

Table S1. Spanish Registry of Pulmonary Arterial Hypertension Centers and Investigators

Hospital Universitario 12 de Octubre - Madrid (Madrid)
P. Escribano
Hospital Universitario y Politécnico La Fe - Valencia (Valencia)
R. López
J. Rueda
Hospital Universitario Insular de Gran Canaria - Las Palmas de Gran Canaria (Las Palmas)
F. Guerra
Hospital Universitario Central de Asturias - Oviedo (Asturias)
P. Bedate
Hospital Universitario Marqués de Valdecilla - Santander (Cantabria)
A. Martínez
Hospital Universitario A Coruña - A Coruña (A Coruña)
I. Otero
Hospital General Universitario de Valencia - Valencia (Valencia)
G. Juan
Hospital Universitario Vall d´Hebrón - Barcelona (Barcelona)
A. Román
Hospital Universitario Virgen del Rocío - Sevilla (Sevilla)
F. García
T. Elías
Hospital Universitario Puerta de Hierro - Majadahonda - Majadahonda (Madrid)
J. Segovia
Hospital Universitario Miguel Servet - Zaragoza (Zaragoza)
J. Domingo
M. López
Hospital Virgen de la Salud - Toledo (Toledo)
M. Lázaro
Hospital Universitario Son Espases - Palma de Mallorca (Islas Baleares)
E. Sala
Hospital Universitario Clínic de Barcelona - Barcelona (Barcelona)
J. Barbera
Hospital Universitario La Paz - Madrid (Madrid)
S. Alcolea
Hospital El Bierzo - Ponferrada (León)
J. Ortiz
Hospital Universitario Basurto - Bilbao (Bilbao)
F. Mazo
Hospital General Universitario Los Arcos del Mar Menor - San Javier (Murcia)
F. Martínez
Hospitales Universitarios Vall d'Hebron - Sant Pau - Barcelona (Barcelona)
L. Dos
Hospital Universitario de Canarias - San Cristobal de la Laguna (Santa Cruz de Tenerife)
A. Lara
Hospital General Universitario de Alicante - Alicante (Alicante)
J. Gil
Hospital del Mar - Barcelona (Barcelona)
L. Molina
Hospital Universitario Ramón y Cajal - Madrid (Madrid)
E. Garrido-Lestache
Hospital Universitario Fundación Jiménez Díaz - Madrid (Madrid)
I. Hernández
